# Transcranial Direct Current Stimulation to Enhance Cognitive Impairment in Parkinson's Disease: A Systematic Review and Meta-Analysis

**DOI:** 10.3389/fneur.2020.597955

**Published:** 2020-11-30

**Authors:** Diana M. A. Suarez-García, Johan S. Grisales-Cárdenas, Máximo Zimerman, Juan F. Cardona

**Affiliations:** ^1^Instituto de Psicología, Universidad del Valle, Santiago de Cali, Colombia; ^2^Institute of Cognitive and Translational Neuroscience (INCyT), INECO Foundation, Favaloro University, Buenos Aires, Argentina

**Keywords:** Parkinson's disease, transcraneal electric stimulation, neuroplasticity, executive functions, cognition

## Abstract

Cognitive deficits are increasingly being recognized as a common trait in Parkinson's disease (PD). Recently, transcranial direct current stimulation (tDCS) has been shown to exert positive effects as an adjunctive therapy on motor and non-motor symptoms in PD. This systematic review and meta-analysis aims to provide an overview of reported evidence on the efficacy of tDCS interventions in the treatment of cognitive impairments in PD. A systematic literature review was conducted to examine articles that were published in the past 10 years and that study the effects of tDCS on cognitive deficits in PD patients. The PubMed, Scopus and Scielo databases were searched. Eight tDCS studies involving 168 participants were included for the analysis. Our meta-analysis results showed that anodal tDCS (atDCS) had various levels or no evidence of effectiveness. In the pre-post stimulation analysis, a strong effect was reported for executive functions (pre-post: *g* = 1.51, *Z* = 2.41, *p* = 0.016); non-significant effects were reported for visuospatial skills (pre-post: *g* = 0.27, *Z* = 0.69, *p* = 0.490); attention (pre-post: *g* = 0.02, *Z* = 0.08, *p* = 0.934), memory (pre-post: *g* = 0.01, *Z* = 0.03, *p* = 0.972) and language (pre-post: *g* = 0.07, *Z* = 0.21, *p* = 0.832). However, in the pre-follow-up stimulation analysis, the duration of the effect was not clear. This study highlights the potential effectiveness of atDCS to improve cognitive performance in PD patients but failed to establish a cause-effect relationship between tDCS intervention and cognitive improvement in PD. Future directions and recommendations for methodological improvements are outlined.

## Introduction

There is growing interest in the potential efficacy of transcranial direct current stimulation (tDCS) for treating neurodegenerative conditions such as Parkinson's disease (PD). Previous systematic reviews on PD have supported the efficacy of tDCS for improving motor functions, including balance, gait, and bradykinesia (1–5). However, evidence is not clear regarding its efficacy for PD patients' cognitive symptoms.

Cognitive impairment is frequent in PD, though it can be heterogeneous in its presentation and progression, as it varies regarding clinical features, severity, and progression to dementia. It has been suggested that interventions for cognitive symptoms may be essential in preventing and delaying the onset of cognitive decline and Parkinson's disease dementia (PDD) (6, 7). Approximately 25% of PD patients have mild cognitive impairment (MCI) and an increased risk of developing PDD (8). Most commonly, reported cognitive disorders in PD include executive deficits (9), visuospatial impairments (10), memory deficits (11), action verb, and action conceptualization impairments (12, 13). These can be progressive and make patients more vulnerable to the onset of affective symptoms, behavioral disorders, and other neuropsychiatric symptoms (14).

tDCS is a non-invasive brain stimulation technique modulating cortical activity that acts by inducing a low-frequency electric current (15), usually between 1 and 2 milliamps (mA), to activate the potential of the resting neuronal membrane (16, 17). The current transmission modifies the membrane's polarity (18), producing a facilitating effect when the positive electric current (anodal) is administered or hyperpolarization when the negative electric current (cathodal) is administered (19).

Given the increasing use of tDCS in neurodegenerative diseases such as PD, the present study aimed to systematically review and analyze studies evaluating the effects of tDCS on PD patients' cognitive alterations.

## Materials and Methods

A systematic literature search was conducted for articles on the effect of tDCS interventions on PD patients' cognitive symptoms. PubMed, Scopus, and Scielo databases were searched for articles published between 2000 and 2020, without language restrictions, combining the following terms: “tDCS,” “transcranial direct current stimulation,” “non-invasive brain stimulation,” and “Parkinson's disease.” We also conducted cross-reference searches of original articles and reviews to identify additional studies that could not be retrieved from electronic databases.

### Inclusion Criteria

This study follows the Preferred Reporting Items for Systematic Reviews and Meta-Analysis (PRISMA) guidelines (20).

### Eligibility Criteria

We used the following PICOT criteria (population, intervention, comparison outcome, and study type) to define eligibility criteria (see Supplementary Material):

- Population: PD and MCI PD patients without dementia diagnosed following UKBB criteria in levodopa on/off stage;
- Intervention: studies evaluating tDCS effects on cognitive functions;
- Comparison outcome: scores obtained on cognitive measures and standard deviation/error.
- Study type: randomized studies with double/single-blind design.

**Figure 1:**
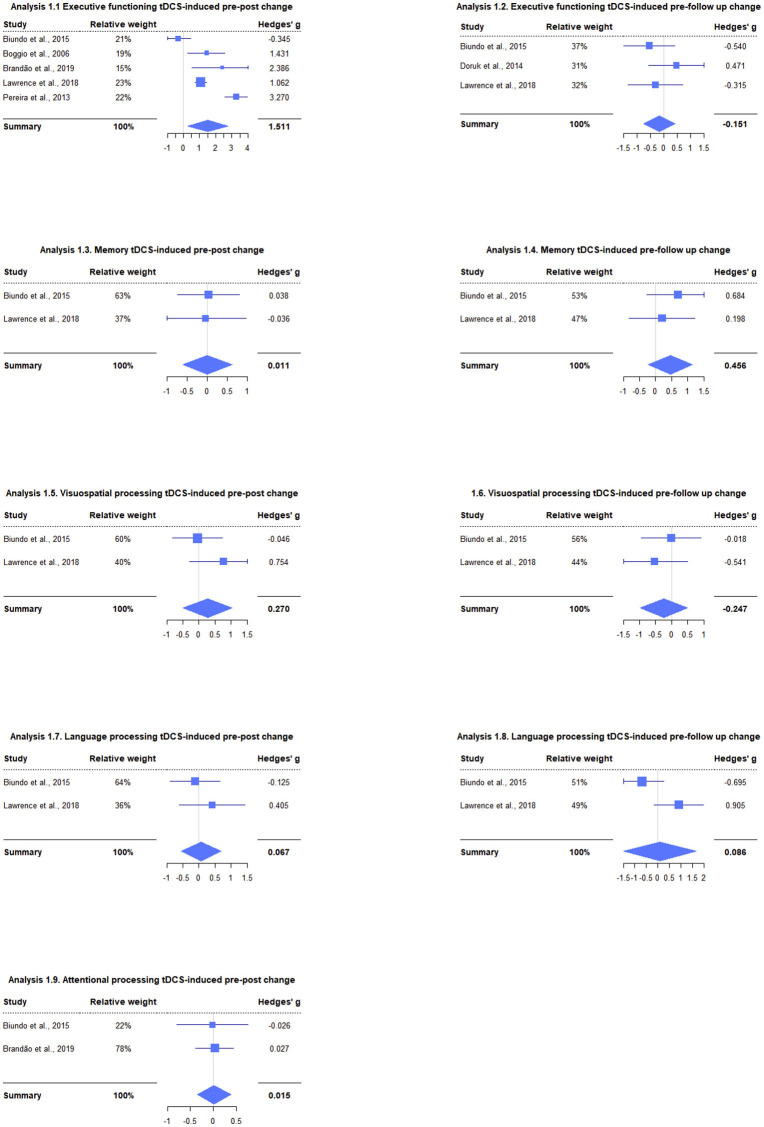
Meta-analyses performed in different cognitive domains for two time points showing both each study effect size and their relative weight within the summary effect size. Effect sizes are expressed in Hedges' g, and the forest plots represent the weight of the studies by the size of the squares, their effect size by their position relative to the x-axis and Hedges' g 95% CI by the squares' lateral bars.

Studies in which data from pre-defined outcomes could not be extracted were excluded (see Figure 1). The following studies were also excluded: (a) animal studies, (b) studies combining tDCS and transcranial magnetic stimulation (21), (c) case studies (22), and (d) non-cortical stimulation studies (23, 24).

### Data Analysis

Several meta-analyses of tDCS vs. sham on cognitive processing was performed following the procedures outlined by Borenstein et al. (25). Interventions' effect sizes were estimated through mean, standard deviation, and sample size. When it was not possible to extract the data, a web calculator was used (26). Because Cohen's “d” overestimates the effect size with small samples, Hedges' “g” was used to correct this bias (27), discriminating between small (0–0.20), medium (0.50–0.80), and large (>0.80) effect sizes (28). Additionally, a random effects approach was used, given its usefulness when there are different designs and response variables. For each analysis, a *z*-test was performed to derive a summary *p*-value. Lau et al.'s (29) study was excluded since needed data for effect-size calculation could not be extracted, while a social cognition meta-analysis could not be performed because Adenzato et al.'s (30) study was the only one to provide such measure.

The cognitive domains were defined according to the characteristics of each study as follows:

- Report of an index or subscale.
- If there were several tasks associated with the same domain, the tasks most used in clinical practice and research were included.
- In the case of a single task/subtest, its effect size was used as the index of the domain to which it was associated.

Meta-analyses were performed at two time points: (a) pre-stimulation to post-stimulation, and (b) pre-stimulation to follow-up. Additionally, as many studies combined tDCS with cognitive training (CT), task scores in interventions that combined stimulation with standard (non-tailored) CT were preferred over task scores in tDCS-only interventions.

### Outcome Variables

As primary outcomes we considered: (1) Measures of executive functions: Problem-solving strategies: The Stockings of Cambridge (SOC) subtest of CANTAB (31); Task-Switching: the Wisconsin Card Sorting Test (WCST), the Trail Making Test B (TMT-B) (32, 33); working memory: the Three-back letter task (34), Visual working memory (VWM), the change detection task (29), the working memory test (WM) (33); inhibition: Stroop Test (Color-word interference) (31–33); verbal and phonological fluency: the Verbal Fluency Test (32), the Controlled Oral Word Association Task (COWAT) (31) and tasks of semantic and phonological fluency (35); (2) Measures of visual attention: the TMT-A (32), the number-letter sequence (LNS) (31); (3) Measures of memory: the Hopkins Verbal Learning Test-Revised (HVLT-R) Immediate recall test, the Paragraph Recall Test (31); (4) Measures of visuospatial skills: the Line Orientation Judgment Test (JLO) and Hooper's Visual Organization Test (HVOT) (31); (5) Measures of language: the Boston Naming Test-Short Form (BNT), the similarity test (31); (6) Measures of theory of mind: the Reading the Mind in the Eyes task, the Attribution of Intentions (AI) task (30); (8) Measures of procedural learning: Probabilistic Classification Learning (PCL) (33); (9) Measures of the inhibition of emotional response: the emotional go/no-go paradigm (29).

## Results

From the initial 248 search results, 32 relevant publications were identified from databases. Of these, eight articles met the inclusion criteria (**see** **Supplementary Material**). The participants' mean age in these studies was 64.2 ± 3.1 years (min 61–max 69). With a total of 168 subjects, the average size of the groups was 21 (10 min and 42 max). The average disease duration and the L-dopa effect were not reported in all the studies.

Overall, 87.5% of the studies reported better cognitive performance after atDCS (see Table 1). Boggio et al. (34) administered 1 and 2 mA atDCS in the left motor cortex (anodal L-M1) or in the left prefrontal dorsolateral cortex (L-DLPFC) with the cathode located in the contralateral supraorbital area (SOAC). They reported high accuracy on the WM, with 2 mA over the L-DLPFC.

**Table 1:** Effect of transcranial direct current stimulation on cognition in Parkinson's disease.

**Study**	**Cognitive abilities**	**Test**	**Total sample** **(*n*)**	**Mean age**	**Evolution of diagnosis**	**On/off** **state**	**Stimulation parameters**					**Results**
							**Active** **electrode**	**Reference electrode**	**Intensity** **(mA)**	**Duration** **(min)**	**Number of** **sessions**	
Adenzato et al. (30)	Theory of mind (ToM)	Reading the mind in the eyes (RME) taskAttribution of intentions (AI) task	(*n* = 20) atDCS (*n* = 20) stDCS	65.6 (8.4)	N/R (MCI)	N/R	MFC (FPz)	Between Inion and Oz	1.5	6	1 atDCS session 1 stDCS session	atDCS over the MFC enhances ToM in patients with PD-MCI.
Biundo et al. (36)	Cognitive functions	MoCA, RBANS Tot., list learning, story learning, complex figure copy, orientation line, naming, semantic fluency, digit span, written coding test, list recall, list recognition, story recall, figure recall	(*n* = 24) (*n* = 12) atDCS (n = 12) stDCS	69.1_7.6	N/R (MCI)	N/R	L-DLPFC	Contralateral supraorbital region	2	20	4 sessions	atDCS over the PFC increased performance in immediate memory skills (story learning test) enhancing declarative and long term memory consolidation.
Boggio et al. (34)	Working memory	Three-back letter working memory paradigm	(*n* = 18) (*n* = 9) atDCS 2mA (*n* = 9) atDCS 1mA	45	Experiment 1 13.7 (8.2) Experiment 2 12.7 (8.1)	OFF	L-DLPFC M1	Contralateral right orbit	Different intensities 1–2	20	2 sessions	2mA of atDCS of the LDLPFC may improve working memory. Beneficial effect on working memory in PD patients depends on the intensity and site of stimulation.
Brandão et al. (32)	Speed processing, executive function, working memory, attention, verbal fluency, inhibitory control	Trail Making Test (TMT), Verbal Fluency test, Stroop test,Timed Up and Go test and video gait analysis.	(*n* = 20) (*n* =10) atDCS (*n* = 10) stDCS	64.45 ± 8.98	7.80 ± 5.32	N/R	L-DLPFC	Right orbital frontal cortex (Fp2)	2	20	1 session	After a single session of tDCS over the DLPFC there is improvements on cognitive tests. Cognitive areas improved the performance in the Stroop test and in the Verbal Fluency.
Doruk et al. (33)	Cognitive functions, depressive symptoms and motor functions	Trail making tests A and B (TMTA and B), Wisconsin card sorting test (WCST), probabilistic classification learning (PCL), working memory test (WM) and stroop test.	(*n* = 18) (*n* =5) atDCS R-DLPFC (*n* = 6) atDCS L-DLPFC (*n* = 7) stDCS	40_71	S/R	ON	L-DLPFC R-DLPFC	Right supraorbital region	2	20	10 sessions	Active stimulation over RDLPFC and LDLPFC resulted in prolonged improvements on executive function (TMT-B test).
Lau et al. (29)	Working memory	Visual working memory task and emotional go/no-go paradigm	(*n* = 10)	56–78	7.8 ± 3.6	ON	L-DLPFC	Contralateral (right) supraorbital area	2	20	1 atDCS session 1 stDCS session	Single-session of atDCS over the L-DLPFC did not significantly improve cognitive tasks in PD
Lawrence et al. (31)	Cognitive function and functional outcomes	Tockings of Cambridge (SOC) subtest from CANTAB and the controlled oral word association task (COWAT), letter-number sequencing (LNS) and the stroop (color-word interference) test, Hopkins verbal learning test-revised (HVLT-R) immediate recall subtest (20) and the paragraph recall test, judgment of line orientation (JLO) test and the Hooper visual organization test (HVOT), y Boston naming test-short form (BNT) and the similarities test.	(*n* = 42) SCT (*n* = 7) TCT (*n* = 7) tDCS (*n* = 7) SCT + tDCS (*n* = 7) TCT + tDCS (*n* = 7) Control (*n* = 7)	SCT: 68.14 (8.69)TCT: 65.57 (5.20)tDCS: 72 6.45SCT + tDCS: 63.57 (15.68)TCT + tDCS: 67.43 (6.37)Control: 72.29 (6.21)	SCT: 5.29 TCT: 5.79 tDCS: 5.50 SCT + tDCS: 6.79 TCT + tDCS: 4.43 Control: 5.36	ON	L-DLPFC	Above the left eye	1.5	20	4 sessions	The intervention groups demonstrated variable statistically significant improvements across executive function, attention/working memory, memory, language, activities of daily living, and quality of life.
Pereira et al. (35)	Phonemic and semantic fluency	Phonemic and semantic fluency tasks	(*n* = 16)	61.5_9.9	S/R	N/R	L-DLPFC L-TPC	Right supraorbital area	2	20	1 session	Functional connectivity in verbal fluency and deactivation task-related networks was significantly more enhanced by tDCS to DLPFC than to TPC. atDCS over l_DLPC increased performance on the phonemic fluency task.

*L-DLPFC, Left dorsolateral prefrontal cortex; R-DLPFC, Right dorsolateral prefrontal cortex; M1, Primary motor cortex; L-TPC, Left temporo-parietal cortex; MFC, Medial Frontal Cortex; MCI, Mild Cognitive Impairment; SCT, Standard Cognitive Training; TCT, Tailored Cognitive Training; tDCS, Transcranial direct current stimulation; atDCS, Anodal transcranial direct current stimulation; ctDCS, Cathodal transcranial direct current stimulation; stDCS, Sham transcranial direct current stimulation; N/R, Not reported*.

Pereira et al. (35) used 2 mA atDCS in the L-DLPFC and left temporoparietal cortex (L-TPC) and cathode in the SOAC. The results showed improvement in phonological verbal fluency after atDCS over L-DLPFC compared to the L-TPC. Additionally, fMRI verified an increase in functional connectivity between the frontal, parietal, and fusiform areas.

Doruk et al. (33) administered 2 mA in the R-DLPFC and L-DLPFC in 18 subjects with PD and located the cathode in the SOAC. The study reports improvement in the TMT-B after bilateral atDCS in the DLPFC.

Biundo et al. (36) used atDCS in the L-DLPFC with 2 mA and placed the cathode in the SOAC in 24 subjects with PD with mild cognitive impairment (MCI-PD). The researchers reported increased immediate memory skills and long-term consolidation of declarative memory.

Lawrence et al. (31) applied atDCS with 1.5 mA in the L-DLPFC and placed the cathode over the left eye in 42 subjects with MCI-PD. The authors implemented various intervention schemes combined with atDCS to assess the impact on cognitive and functional performance. Evidence suggests improvement in executive function, attention/WM, memory, language, daily living activities, and quality of life compared to the control group when combining CT and atDCS.

Adenzato et al. (30) administered 1.5 mA atDCS to the medial frontal cortex (MFC) and placed the cathode between the Inion and Sickle in 20 MCI-PD patients. The authors report a significant correlation between the reaction time (RT) of the Attribution of Intentions (AI) task and the Frontal Assessment Battery (FAB) score and the effect of interference in time and Stroop error. Findings are limited to improvement in RT; no significant improvement in response precision was observed. Researchers suggest that atDCS in MFCs improves deficits in the Theory of Mind (ToM) in MCI-PD.

Brandão et al. (32) investigated the effect of atDCS on executive functions, verbal fluency, and inhibitory control in 20 subjects with PD when administering 2 mA for 20 min in the L-DLPFC. The cathode was placed in the SOAC. The study reports improvement in the performance of cognitive tests STROOP—inhibition and interference—and verbal fluency in the group that received atDCS. The authors do not report a significant difference in the TMT-B or motor measurements.

Lau et al. (29) applied 2 mA to the L-DLPFC in 10 subjects with PD without cognitive compromise, locating the cathode in SOAC. The researchers evaluated VWM and emotional inhibitory control using experimental paradigms. The study suggests that performing a single session of atDCS is insufficient to generate significant VWM and emotional inhibition processes in subjects with PD. However, the authors also highlighted the small sample size.

We ran 2 meta-analyses per cognitive domain: (a) one analyzing the pre-post stimulation period and (b) one analyzing the pre-follow-up stimulation period. Regarding executive functions, the results showed large effects of improvement in performance in the pre-post period and small and non-significant effects in the pre-follow up [pre-post: *g* = 1.51, 95% CI = (0.28, 2.74), *Z* = 2.41, *p* = 0.016; pre-follow up: *g* = −0.15, 95% CI = (−0.75, 0.45), *Z* = −0.50, *p* = 0.619], see Figure 1, analysis 1.1 y 1.2. In memory, there was a medium effect for the pre-follow-up period of improvement in cognitive performance, although it was not significant, while for the other period, the effect was small and non-significant [pre-post: *g* = 0.01, 95% CI = (−0.60, 0.63), *Z* = 0.03, *p* = 0.972; pre-follow-up: *g* = 0.46, 95% CI = (−0.24, 1.15), *Z* = 1.28, *p* = 0.199] (Figure 1, analysis 1.3 y 1.4). The analyses in visuospatial skills showed medium effects with improvement in the pre-post and decrease in performance in the pre-follow up, although neither was significant [pre-post: *g* = 0.27, 95% CI = (−0.50, 1.04), *Z* = 0.69, *p* = 0.490; pre-follow up: *g* = −0.25, 95% CI = (−0.98, 0.49), *Z* = −0.66, *p* = 0.511], Figure 1, **analysis 1.5 y 1.6**. In language, a small and non-significant effect was observed for both time points [pre-post: *g* = 0.07, 95% CI = (−0.55, 0.68), *Z* = 0.21, *p* = 0.832; pre-follow up: *g* = 0.09, 95% CI = (−1.48, 1.65), *Z* = 0.11, *p* = 0.915], Figure 1, analysis 1.7 y 1.8. Finally, for visual attention, a small and non-significant effect was observed [pre-post: *g* = 0.02, 95% CI = (−0.35, 0.38), *Z* = 0.08, *p* = 0.934], see Figure 1, analysis 1.9.

## Discussion

This systematic review has highlighted that there are a limited number of studies examining the effects of tDCS on cognitive outcome measures in PD. The few studies available, suggest that atDCS has a positive effect mainly in executive functions. In this regard, studies have shown better performance in problem-solving tests (31), verbal fluency (35, 36), cognitive flexibility (33), planning, and WM (33, 34). Additionally, two studies highlight greater precision and retention of information in memory tests and procedural learning (35, 36). The meta-analysis converges, highlighting positive effects on executive performance; however, these analyses are small (2–5 studies) and subject to considerable variability, so they should only be taken as exploratory. Similarly, while most results were non-significant, uncertainty around the point estimates was underscored by the wide confidence intervals calculated, further stretching the need for studies to clarify and improve the effect-sizes estimations. Interestingly, variations in the detected effects may arise depending on the time point chosen for assessment, i.e., an effect may remain or disappear in the follow-up, or even appear in the follow-up after not having been detected in the post-treatment measure, which would suggest that some effects are only detected after potential learning effects, masking those that could be attributed to tDCS, have vanished. These findings suggest both the need to control for practice effects and to perform at least one follow-up assessment. Consequently, it is important to fine-tune and standardize the time points for follow-up assessments.

Only one study focused and reported positive effect on electrical activity and functional connectivity circuits in PD (35). It could be speculated that, due to action mechanisms and diffuse effects of tDCS, when applied in frontal areas, this technique increases the electrical activity and functional connectivity of cortico-striatal and thalamocortical circuits (37) affected in PD (38). However, it would be hasty to make this statement without clarity on some methodological aspects and more evidence to support this hypothesis.

Although most studies have used atDCS in the L-DLPFC, some studies do not clarify the neuroanatomical coordinate system used to locate the anode. Thus, it is suggested that future studies verify the correct electrodes' position through mathematical simulation of the electric fields generated by the assembly (39). Moreover, there is variability in current intensity (1–2 mA) and the period of exposure to tDCS, which prevents identifying if effects hold over time. Performing a current stimulation process for a few seconds can generate changes in cortical excitability. However, these are insufficient to consider them significant. Indeed, when stimulation is prolonged or repetitive, effects can last for hours (16, 40) and even days (19). The most widely used stimulation parameters to establish the use of tDCS in PD are 6–20 min per session, and no more than twice per day (41).

Our review and meta-analysis suggest that tDCS has been shown to exert positive effects as an adjunctive therapy on non-motor symptoms in PD. It is not sufficiently evidenced to establish a cause-effect relationship between tDCS intervention, cognitive improvement, electrical activity modulation and functional connectivity increase in PD. Thus, it is essential to (a) explore the potential of tDCS to ameliorate another kind of cognitive symptom reported in PD, such as action verb processing impairment (12, 13, 42–45); to date, there is no evidence about it, and it is feasible to stimulate networks involving cortico-cortical fibers and cortico-subcortical circuits (37) primarily affected in PD (43). It is also essential to (b) perform longitudinal studies to determine whether changes in cognition persist over time. Limited number of sessions and periodicity of the process currently impedes testing whether the effect is transitory and experimentally relevant or if it could go beyond therapeutic and clinical applicability.

### Limitations and Suggestions for Further Research

Several factors limit interpretations of these studies' results and the understanding of tDCS effects on cognitive impairments in PD patients. As mentioned by Borenstein et al. (25), including studies with independent and related groups in the same meta-analysis introduces a source of error to be considered. However, the decision was made due to the limited number of studies; therefore, results should be taken carefully and in an exploratory way. An heterogeneity analysis was not conducted since, as reported previously, for such small analyses this type of test has low statistical power (46, 47).

The lack of standardization of the outcome measures used to assess changes in cognitive performance in different domains, has led to a considerable variability in the analyses performed. This should be addressed in the future by establishing a set of measures that can sensibly evaluate tDCS-related changes. Although results are promising and tDCS is positioning itself as a new adjuvant therapy in PD treatment, sample groups are small and heterogeneous; therefore, it is necessary to conduct studies with larger cohorts. Likewise, it is recommended to combine (a) intervention schemes involving pharmacological treatment and physical and CT programs to determine under what conditions the modulating effect of tDCS is enhanced, and (b) further research should employ neurophysiology measurements to characterize and explore the potential cause-effect relationship between tDCS intervention, cognitive improvement, and neural correlates -as connectivity signatures- in PD.

## Conclusion

This systematic review and meta-analysis highlight potential effectiveness of atDCS to improve executive (including inhibition of prepotent responses, shifting mental sets, monitoring and regulating performance, goal maintenance, planning, working memory, and cognitive flexibility) and mnemonic performance in PD patients but failed to establish a cause-effect relationship between tDCS intervention and cognitive enhancement in PD.

Considering the potential value of this safe and low-cost technique, it is imperative that well-designed, high-quality, and sufficiently powered randomized studies assess the efficacy of tDCS to treat cognitive impairments in PD and draw new pathways to include it in clinical practice. Evidence from the effects of tDCS on cognitive symptoms in PD patients is sparse, and we suggest that further research is required.

## Author Contributions

DS-G, JG-C, MZ, and JC developed the review concept. DS-G, JG-C, and JC drafted the manuscript. MZ provided critical revisions. DS-G and JG-C performed the data collection, analysis, and interpretation under the supervision of JC and MZ. All authors approved the final version of the manuscript for submission.

## Conflict of Interest

The authors declare that the research was conducted in the absence of any commercial or financial relationships that could be construed as a potential conflict of interest.
