# Supplementary figures and images for: Transcranial Direct Current Stimulation to Enhance Cognitive Impairment in Parkinson's Disease: A Systematic Review and Meta-Analysis
Source: Front Neurol. 2020 Nov 30;11:597955. doi: 10.3389/fneur.2020.597955 (PMC7734248; doi:10.3389/fneur.2020.597955)

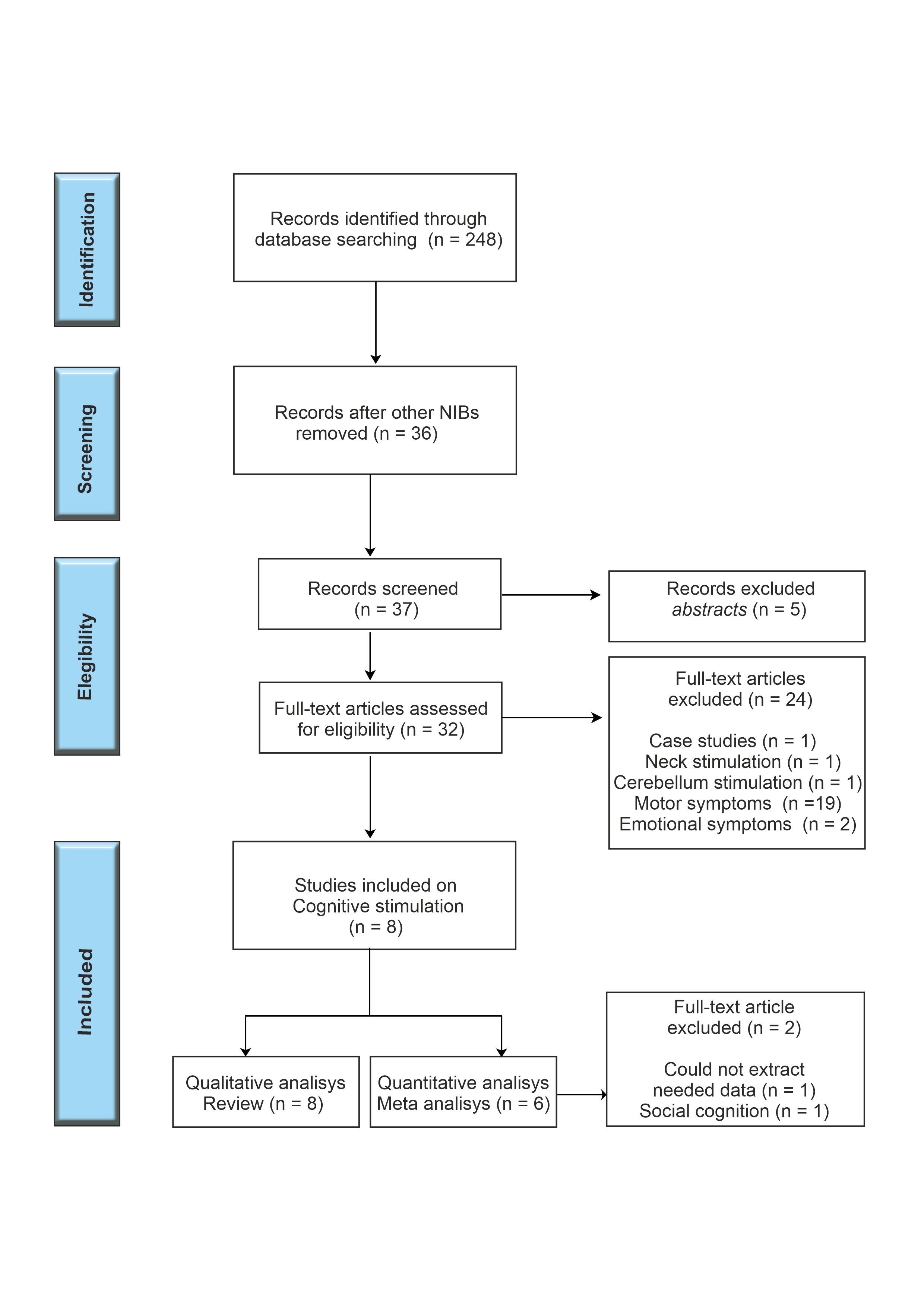

Supplement: Supplementary file 1 [file Image_1.TIF]
